# Pharmacist E‐script transcription service initiated nicotine replacement therapy uptake in pre‐admission clinic: A pilot study

**DOI:** 10.1002/hpja.910

**Published:** 2024-09-02

**Authors:** Darshana Meanger, Ashley Webb, Iouri Banakh, Lisa Coward, Gael Cripps, Johnson George

**Affiliations:** ^1^ Peninsula Health Pharmacy Department and Eastern Health Pharmacy Department Frankston Hospital Frankston Australia; ^2^ Peninsula Health Anaesthetics Department Frankston Hospital Frankston Australia; ^3^ Monash Health Pharmacy Department Frankston Hospital Frankston Australia; ^4^ Peninsula Health Preadmission Clinic Frankston Hospital Frankston Australia; ^5^ Centre for Medicine Use and Safety, Monash University Frankston Australia

**Keywords:** abstinence, nicotine replacement, pharmacist, pre‐admission clinic, smoking cessation

## Abstract

**Background:**

Pharmacist‐led smoking cessation programs in pre‐admission clinics (PAC) have shown to increase quit attempts and achieve abstinence by the day of surgery (DOS).

**Aims:**

To evaluate the feasibility of Pharmacist E‐script Transcription Service (PETS) initiated nicotine replacement therapy (NRT) in PAC, including smoking cessation on DOS.

**Methods:**

A single centre, pre and post‐intervention pilot study conducted at an Australian public hospital PAC. In a two‐month intervention period, PAC nursing staff invited smokers (≥1 cigarette/day) to see a smoking cessation PET pharmacist. Pharmacist‐initiated NRT and Quitline© referrals were offered. Cessation outcomes were compared with the preceding two‐month control period. Primary outcome: feasibility of intervention. Secondary outcomes: DOS smoking abstinence rates and three‐months post‐surgery.

**Results:**

PAC nurses identified 112 smokers over 4 months; 53 during pre‐intervention period, and 59 during intervention period. Twenty‐two intervention patients (37%) accepted seeing the pharmacist, with 16 subsequent Quitline© referrals (73%) and 11 NRT prescriptions (50%) written. The median nursing smoking status documentation time increased in the intervention period (1 min vs. 4, *p* < .001). The intervention did not impact pharmacist's workload. Verified abstinence increased from 8.5% (4/47) pre‐intervention to 9.4% (5/53) post‐intervention, *p* =1.00. Relapse rates in the intervention period increased (20% vs. 50%) at three‐months post‐surgery.

**Conclusion:**

A PETS‐initiated NRT program in PAC is feasible and increased preoperative use of NRT and Quitline© with minimal impact on smoking cessation.

**So what?:**

This study has highlighted the importance of implementing a multidisciplinary smoking cessation program in PAC however, larger studies are needed to determine the true impact of the program on smoking cessations.

## 1 INTRODUCTION

Smoking increases risks of post‐surgical complications, including post‐operative wound infections and respiratory complications; on the other hand, pre‐operative quitting for 4–8 weeks significantly decreases these risks.^1^, ^2^

Several studies of smoking cessation interventions in pre‐operative clinics have been published, with most providing behavioural counselling and/or nicotine replacement therapy (NRT).^3^, ^4^, ^5^, ^6^ While pre‐admission clinics (PAC) were shown to be feasible locations for offering smoking cessation interventions, with up to 60% of smokers accepting help, many of the published studies utilised resource intensive, high intensity interventions that may lack feasibility in routine clinical practices.^3^, ^7^ Programs that utilise existing and funded services such as Quitline© and the Pharmaceutical Benefits Scheme (PBS) to reduce perioperative smoking are more likely to be implemented in resource constrained health system. In 2023 the general patient cost of 28 21 mg/h nicotine patches under the PBS was $30 compared to the non‐subsidised cost of $60.^8^, ^9^ Smoking cessation best practice involves behavioural support in combination with pharmacotherapy.^9^ Quitline© is a free call‐back behavioural support service and is a pre‐requisite for subsidised NRT prescriptions; NRT prescription recipients are asked to enrol in Quitline©.^8^

Pharmacist‐led smoking cessation interventions have shown to be effective in achieving both short‐ and long‐term abstinence in pre‐operative, hospital inpatient and rehabilitation settings.^10^, ^11^, ^12^, ^13^ Additionally, many health services have established Pharmacist E‐script Transcription Service (PETS) or similar services with trained pharmacists to co‐prescribe/transcribe discharge prescriptions. While prescriptions are required to be co‐signed by the treating doctor, the PETS model has been shown to facilitate the discharge process and reduce prescribing errors.^14^ Utilisation of this PETS model to increase provision of PBS prescriptions of NRT to outpatients is novel and may reduce existing barriers to the uptake of NRT in the PAC,^7^ such as lack of time or knowledge of NRT prescribing by junior medical staff.

This study evaluated the feasibility of a PETS‐initiated NRT program in PAC and compared the effects on smoking abstinence rates by day of surgery (DOS) against standard care in a smoke‐free public hospital.

## 2 METHODS

### 2.1 Setting and participants

Single centre, pre‐ and post‐interventional pilot study at Frankston Hospital PAC, a smoke‐free tertiary care public hospital in Victoria, Australia. The study included all adult elective surgery patients attending Frankston Hospital PAC (March–June 2021) who self‐reported being current smokers (≥1 cigarette/day). Exclusion criteria were inability to communicate in English, understand study requirements, surgery dates scheduled for the weekend or at a private hospital (due to lack of data collector availability).

### 2.2 Study design/intervention

Patients in the two‐month standard care group (pre‐intervention period) received no systematic smoking cessation pharmacotherapy or Quitline© support in PAC. Pre‐intervention, patients attending PAC saw a variety of different clinicians (nurses/surgical residents or registrars/anaesthetist/physiotherapist/pharmacist) with no single clinician having primary responsibility to address patient smoking and provide cessation advice/help. Smoking cessation brochures were available in PAC, but provision was variable depending on clinician. Pharmacist in the pre‐intervention period only had capacity to see high risk peri‐operative patients due to limited resources.

Prior to the two‐month intervention period, pharmacists and PAC nurses undertook free online Quit Victoria ‘Brief advice training’ to improve counselling skills.^15^ During the intervention period, PAC nurses systemically documented smoking status, provided structured quit smoking advice before surgery and invited patients to see a PETS PAC pharmacist for further cessation assistance. Those agreeing for pharmacist consultation were offered a Quitline© referral. Participants that signed up to Quitline© were offered a pharmacist generated PBS prescription for NRT (unless contraindicated).

Pharmacists prescribed NRT electronically (CernerCorp, North Kansas City, USA) using pre‐populated ‘Power Plans’, according to patient's nicotine dependence (low, medium, or high). As standard practice, the PETS NRT prescriptions were later co‐signed by surgical staff. Smokers were advised to consult their general practitioner (GP) or community pharmacist for additional NRT beyond the initial script. PAC nurses systematically provided the Peninsula Health ‘Stop Smoking’ leaflet to all PAC smokers.

On DOS (prior to operation), cessation data was collected by researchers using a structured questionnaire. If claiming quit >24‐h, verification with carbon monoxide (CO) breath testing (quit ≤8 parts‐per‐million (ppm)) was requested and quit patients were telephoned three‐months post‐discharge to determine smoking relapse. Those who refused a CO breath testing or those showing >8 ppm was classified as current smoker.^16^ Seven‐day point prevalence was collected to account for those that had less than 7 days to quit due to their PAC appointment being close to their surgery.

### 2.3 Consent

Research assistance obtained a verbal consent to undertake the DOS survey and a written consent for a CO breath test and three‐month follow up.

### 2.4 Measures and tools

Acceptability was measured as the proportion of patients agreeing to see the pharmacist after invitation by PAC nursing staff, and the rate of patients agreeing to NRT/Quitline© prescription by the pharmacist. Number of Quitline© contacts by DOS and at three‐months was also collected during participant follow up.

Heaviness of smoking index (HSI) score is a scoring tool that utilises two questions from the Fagerstrom Test for Nicotine Dependence (time to first cigarette and number smoked per day).^17^, ^18^ The HSI score ranges from 0 to 6 (low (0–1), medium (2–4) or high (5‐6)). All patients' HSI scores were assessed at PAC and on the DOS.^18^

Naranjo Algorithm is an Adverse Drug Reaction (ADR) probability scale that assesses if the ADR has a causal relationship to the suspected treatment.^19^ This scale was utilised as part of the DOS interview when assessing NRT related ADRs.

Visual analogue scale (VAS) was used to measure the patients' level of confidence and motivation to quit smoking. This 10‐point numerical scale ranges from zero for ‘not motivated or confident’ to 10 for ‘very motivated or confident’.^20^

### 2.5 Outcome measures

1. Primary outcome measures for feasibility were acceptability (percentage that accepted the intervention), demand (percentage eligible to see the pharmacist and for a NRT prescription), implementation (change in PAC workflow), practicality (time taken for nurses to address smoking cessation and number of patients seen by the pharmacist) and adaptation (percentage of PAC staff who underwent Quitline© training and education).
2. Secondary outcome measures were abstinence rates (quit for >24 h with a CO level of ≤8 ppm), self‐reported reduction in smoking, reduction in HSI scores by the DOS, > 7 day quit rates, NRT and Quitline© uptake and abstinence at three‐months post‐surgery in both periods.

### 2.6 Statistical analysis

Data analysis was performed using Statistical Package for Social Sciences (SPSSversion 25). Mann–Whitney U tests were used to compare feasibility outcomes pre and post‐intervention (time taken for PAC staff to complete data collection sheets and the number of patients seen by the pharmacist in PAC). The standard deviation of the mean times of each nurse in the pre‐intervention period was calculated to determine the variability in the time taken to address smoking cessation between the nurses. Those who declined to be followed‐up on the DOS were treated as lost to follow up and were excluded from the analysis. Fisher's exact test was used to analyse DOS secondary outcomes including self‐reports of abstinence, biochemically verified self‐reports of abstinence, reduction in HSI score as well as continuous abstinence at three‐months post‐surgery. Those that refused to undertake the CO breath test on the DOS were treated as smoking abstinence failures and were included in the final analysis. An unpaired student *t*‐test was used to analyse continuous data (e.g., age) and a Chi‐square test was used to analyse categorical data (e.g., sex, level of nicotine dependency and category of surgery). Categories of surgeries were defined based on the urgency of the surgery (category 1 within 30 days, category 2 within 90 days and category 3 within a year). A sample size calculation was not undertaken given that it was a pilot study to determine feasibility of the intervention.

## 3 RESULTS

**FIGURE 1:**
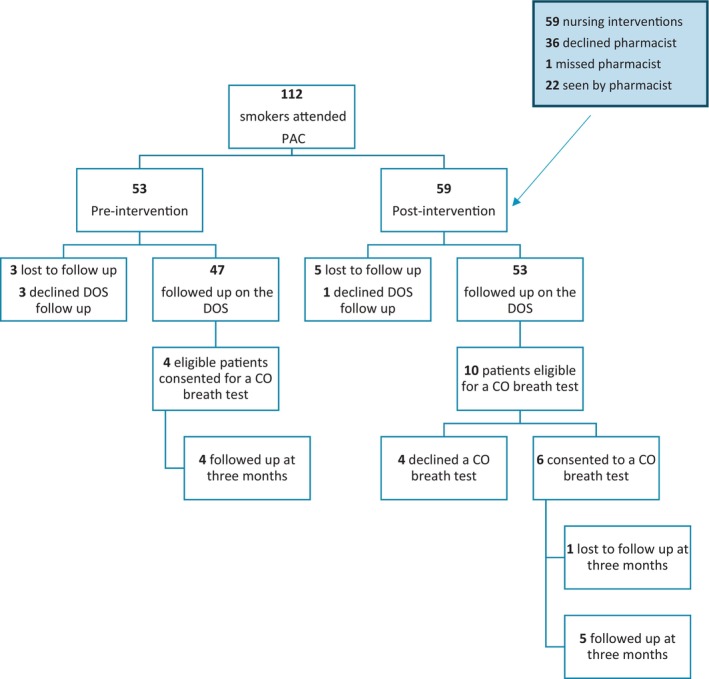
Patient flow.

A total of 640 patients were scheduled to attend the PAC between March to July 2021, of which 582 patients were screened for study eligibility (Figure 1). Fifty‐eight patients were not screened due to failed appointment attendance or incomplete data collection sheets. Eight patients were lost to follow‐up on the DOS due to either the COVID‐19 pandemic, weekend surgery dates, private hospital admission or missed by researchers.

The population screened had a 19.2% (112/582) smoking rate with 53 patients in the pre‐intervention group and 59 patients in the intervention group (Figure 1). Patient characteristics were overall similar in both periods, but there were more category 3 and orthopaedic surgeries in the pre‐intervention group, while urgent general surgery were more frequent in the intervention group (Table 1).

**TABLE 1:** Baseline characteristics of participants.

Characteristics	Pre‐intervention (*n* = 53)	Intervention (*n* = 59)	*p*‐Value
Gender			
Female	**25 (47%)**	25 (42%)	.704
Male	28 (53%)	**34 (58%)**	.610
Age mean in years (SD)	**62.8 (11.8)**	59.6 (12.6)	.303
Surgery category			
Cat 1	21 (40%)	**22 (37%)**	.847
Cat 2	17 (32%)	**33 (56%)**	**.014**
Cat 3	**15 (28%)**	4 (7.0%)	**.005**
Pension card	**36 (68%)**	34 (58%)	.329
HSI score			
Low (0–2)	31 (58%)	**40 (68%)**	.332
Medium (4‐3)	**20 (38%)**	17 (29%)	.421
High (5, 6)	**2 (4.0%)**	2 (3.0%)	.912
PAC to OT time			
< 7 days	14 (26%)	**16 (27%)**	.933
7–32 days	29 (55%)	**33 (56%)**	.897
> 32 days	**10 (19%)**	10 (17%)	.810

*Note*: Bold indicates a higher comparative number.

Abbreviations: HSI, Heaviness of Smoking Index; OT, operating theatre; PAC, Pre‐admission clinic; SD, standard deviation.

### 3.1 Primary outcomes

In the intervention period 39% (23/59) patients agreed to see the pharmacist, with one low dependency patient refusing to wait for the pharmacist. Accepted patients were highly motivated to quit (median score of 8 (IQR =3)) and confident of success (median score of 7 (IQR = 4)) but high levels of nicotine dependence were uncommon (Table 2). Out of the 22 patients, 16 (72%) were signed up to Quitline© and 11 (50%) requested and were generated a PETS NRT prescription. Three patients were ineligible for NRT prescription as they were already using stop smoking medications or products (e‐cigarettes and NRT) at the time of their PAC appointment.

**TABLE 2:** Characteristics of patients who accepted to see the pharmacist.

Characteristics of acceptors	*n* = 22^a^
Level of dependency (HSI score)	
Low (0–2)	9 (40%)
Medium (4, 5)	12 (52%)
High (5, 6)	1 (4.5%)
Motivation to quit	
Low (0–5)	2 (9%)
High (6–10)	20 (91%)
Confidence to quit	
Low (0–5)	7 (32%)
High (6–10)	15 (68%)
NRT prescribed	
Patch	5 (23%)
Lozenge	2 (9%)
Combination	4 (18%)

^a^ Minus one acceptor who did not wait to see the pharmacist (Total 23 acceptors).

Three reports of workflow issues and one report of prescription signage delay were identified. As expected, nursing staff spent more time discussing smoking cessation in the post‐intervention period then the pre‐intervention period (Table 3). Smoking status documentation improved from pre‐ to post‐intervention through completing of the PAC tobacco cessation tool. Pharmacists in the pre‐intervention period only had capacity to see high‐risk peri‐operative patients due to limited resources.

**TABLE 3:** Time taken by nursing staff and number of patients seen by the pharmacist in PAC.

Measures	Pre‐intervention	Intervention	*p*‐Value
PAC nursing staff time taken in minutes (median (IQR))	1.0 (1.0)	4.5 (3.0)	<.001
No. of patients seen by the pharmacist (median (IQR))	6.0 (3.0)	6.0 (2.0)	

Abbreviations: IQR, Interquartile range; OT, operating theatre; PAC, Pre‐admission Clinic.

The workload of the pharmacist was not increased by the intervention deeming the intervention feasible (Table 3).

### 3.2 Secondary outcome

Biochemically (CO breath test)‐verified self‐reported abstinence rates slightly improved pre and post‐intervention (4/47 vs. 5/53) however self‐reported abstinence rates doubled from 8.5% (4/47) in the pre‐intervention period to 18.9% (10/53) in the post‐intervention period (*p* = .152) (Table 4). One patient in the post‐intervention period recorded a CO level of 25 ppm. Seventy‐one percent (38/53) of patients in the post‐intervention group self‐reported to have cut down by the DOS compared to 64% (30/47) in the pre‐intervention group (*p* = .520). A larger number of patients with a reduction in their HSI score on the DOS was seen in the post‐intervention group (Table 4).

**TABLE 4:** DOS smoking characteristics.

Measures	All patients			Seen by pharmacist		
	Pre‐intervention (*N* = 47)	Post‐intervention (*N* = 53)	*P*‐Value	Pre‐intervention (*N* = 20)	Post‐intervention (*N* = 22)	*P*‐Value
Self‐reported DOS quit rates						
7 day point prevalence	2 (4.2%)	**3 (5.7%)**	1.000	1 (5.0%)	**2 (9.0%)**	1.000
< 7 day point prevalence	2 (4.2%)	**7 (13.2%)**	.162	0 (.0%)	**3 (13.6%)**	.233
Total	4 (8.5%)	**10 (18.9%)**	.152	1 (5.0%)	**5 (22.7%)**	.187
Biochemically verified DOS quit rates						
7 day point prevalence	**2 (4.2%)**	2 (3.8%)	1.000	**1 (5.0%)**	1 (4.5%)	1.000
< 7 day point prevalence	2 (4.2%)	**3 (5.7%)**	1.000	0 (0.0%)	**1 (4.5%)**	1.000
Total	4 (8.5%)	**5 (9.4%)**	1.000	1 (5.0%)	**2 (9.0%)**	1.000
DOS smoking characteristics						
Self‐report cut down	30 (63.8%)	**38 (71.0%)**	.520	15 (75.0%)	15 (68.0%)	.738
Reduction in HSI score	11 (23.4%)	**15 (28.0%)**	.651	3 (15.0%)	**8 (36.0%)**	.165
No. using NRT	12 (25.5%)	**19 (34.0%)**	.287	7 (35.0%)	**10 (45.0%)**	.543
No. contacted quitline	4 (8.5%)	**14 (26.0%)**	.035	1 (5.0%)	**10 (45.0%)**	.004
Perioperative quit attempt (ended in relapsed)	4 (8.5%)	**8 (15.0%)**	.368	1 (5.0%)	**2 (9.1%)**	1.000
Using stop smoking medications	16 (34.0%)	**20 (38.0%)**	.835	8 (40.0%)	**10 (45.0%)**	.764

*Note*: Bold indicates a higher comparative number.

Abbreviations: DOS, Day of Surgery; HSI, Heaviness Smoking Index; NRT, Nicotine Replacement.

DOS NRT use (34% vs. 25.5%) and Quitline© contact (26% versus 8.5%) was higher in the post‐intervention group compared to the pre‐intervention group (Table 4). One patient given an NRT lozenge prescription experienced an ADR (heart racing).

Post‐operative relapse to smoking was common with two of the four verified quitters in the pre‐intervention group (50%) and four of the five verified quitters (80%) in the intervention period relapsing. NRT use remained high in the intervention group post‐surgery (80% [4/5] vs. 25% [1/4], respectively, *p* = .206).

## 4 DISCUSSION

Smoking cessation interventions in PAC such as the PETS initiated NRT program is feasible to implement and affordable when leveraging off existing funded programs such as the PBS and Quitline©. This pilot study of a pharmacist‐led smoking cessation program in PAC demonstrated some favourable trends such as: increase in NRT uptake and reduction in HSI scores but did not change biochemically verified abstinence.

With acceptance rates mostly compromising of low to moderate nicotine dependent participants this study further highlights the ongoing difficulty in engaging high dependency smokers due to minimal motivation and confidence to quit in these settings. This indicates that ‘brief’ perioperative smoking cessation advice may not be enough for high dependency smokers. Close to half of the patients who accepted to see the pharmacist in intervention period were of low dependency, for whom nicotine replacement therapy is not necessarily needed. Highlighting that future models of care could consider having nursing staff manage low dependency patents, allowing the pharmacist to maintain their focus on moderate‐high dependency patients. Interestingly, a larger number of patients reported to be using NRT on the DOS in the post‐intervention period than prescribed in the PAC, highlighting that just the conversation in PAC may influence NRT uptake before the DOS.

Three in five patients refused seeking pharmacist assistance for smoking cessation support, despite impending surgery. Acceptance rates of the intervention were like other studies that offered behavioural support and NRT.^4^ However, most studies provided free pharmacotherapy which may not be feasible and/or did not have a pharmacist‐led model of care.^4^, ^21^, ^22^, ^23^, ^24^, ^25^ Surgeon‐led interventions have shown to increase the desire to quit before surgery.^19^ Patient pharmacist acceptance rates for smoking cessation support could be increased through referrals from other health professionals in PAC such as doctors, an ‘opt out’ not ‘opt in’ to see the pharmacist process or use of CO breath tests to increase interest in quitting.

This study demonstrated similar improvement in self‐reported abstinence rates by the DOS compared to other studies.^23^ However studies such as Lee et al had shown a greater improvement in biochemically verified self‐reported abstinence rates, potentially due to a higher CO level cut off (<10 ppm versus <8 ppm).^4^ The study showed that patients who were seen by the pharmacist in PAC were more likely to have self‐reported abstinence, NRT use and Quitline© uptake. The difference between self‐reported smoking reduction rates pre‐ and post‐intervention in those who had been seen by the pharmacist were better than that of Lee et al (20.6% versus 32.5%); however, the results failed to achieve statistical significance.^4^

The study had a much higher relapse rate at three‐months post‐surgery despite an increase in NRT/Quitline© use. Possibly due to an increase in stressors during the COVID pandemic and lack of motivation to quit for life. Suggesting that newly quit smokers may need more support post‐surgery to encourage continuous abstinence such as ‘relapse packs’ offered by Web et al.^25^

### 4.1 Strengths and limitations

The study used a novel multidisciplinary structured pharmacist‐led smoking cessation program, which utilised the RACGP recommended AAH model of care and Quitline© behavioural support.^26^ This model of care can be easily implemented into any healthcare system, given the use of PBS to minimise any additional funding requirements. This study expanded the scope of pharmacists and has shown that a pharmacist‐led model of care can be feasible for larger, future studies.

The study's major limitation was the intervention's implementation during the COVID‐19 lockdowns leading to a major difference in the pre‐post period. This led to changes in categories and types of surgeries between the two study periods due to changes in elective surgery criteria. Furthermore, the COVID‐19 outbreak may have discouraged patients from considering quitting given the added stressors leading to a decline in acceptance rates during this period and an increase in smoking relapse rates at three‐months post‐surgery in the post intervention period. However, the study did not capture the confidence data in the pre‐intervention period therefore there is no way to verify this. Despite the excess demands on the hospital during the COVID‐19 lockdown, the pharmacists saw a similar number of patients as the pre‐intervention period, highlighting the intervention model's viability. Other limitations of the study included the single‐centre and non‐parallel study design, which may have resulted in selection bias. Group sizes were small and most patients who had accepted the invitation to see the pharmacist were very confident and motivated to quit. Baseline group characteristics were similar, although there was a small difference in nicotine dependence, which may have had an impact on overall quit rates.

Self‐reports may have led to social desirability bias when responding to questions. CO breath test was utilised to biochemically verify self‐reports on the DOS to objectively verify self‐reported abstinence. However, biochemical verification was not possible at three‐month follow up due to limited resources. Lastly, the HSI score can have a floor effect amongst light smokers which may impact the validity of the tool in this population. HSI score does not consider the number of quit attempts which if taken into account could alter the number of participants who were of ‘low nicotine dependence’.

## 5 CONCLUSION

A PETS initiated NRT program in PAC is a novel cost effective and feasible initiative that led to increased perioperative NRT uptake, increase Quitline© use, reduce nicotine dependency, and minor improvement in quit rates. The program has now been implemented into routine presurgical care. Further studies are needed to quantify the improvement in abstinence rates by the DOS observed; Economic evaluation of the intervention will be valuable to inform resource allocation and policy making. In addition, there is a gap in research involving pharmacist‐led models of care in a preoperative clinic setting and more studies are needed to be able to determine true effect on both short and long‐term abstinence of such a model in clinical practice. This study has highlighted the importance of implementing a multidisciplinary smoking cessation program in PAC to encouraging elective surgery patients to begin their quit journey.

## CONFLICT OF INTEREST STATEMENT

The authors declare no conflicts of interest.

## ETHICS STATEMENT

The Peninsula Health Research Ethics Committee approved the study and waived the requirement for consent by participating patients (HREC/70155/PH‐2020).

## Data Availability

The data supporting the findings of this study are available on request from the corresponding author. The data are not publicly available due to privacy or ethical restrictions.
